# Crude annual incidence rate of medullary thyroid cancer and *RET* mutation frequency

**DOI:** 10.3325/cmj.2021.62.110

**Published:** 2021-04

**Authors:** Sara Milićević, Damijan Bergant, Tina Žagar, Barbara Perić

**Affiliations:** Institute of Oncology Ljubljana, Ljubljana, Slovenia

Medullary thyroid carcinoma (MTC) represents 5%-10% of all thyroid cancers (1). It occurs in either sporadic (75%) or hereditary form (25%) as a part of multiple endocrine neoplasia type 2 syndrome (MEN 2). MEN 2 results from an autosomal dominant, missense, gain-of-function mutation in the rearranged during transfection (*RET*) proto-oncogene (2). The syndrome has two subtypes: MEN 2A and MEN 2B. MEN 2A is characterized by MTC (95%), pheochromocytoma (30%-50%), primary hyperparathyroidism (10%-20%), Hirschsprung's disease (7%), and in rare cases cutaneous lichen amyloidosis (3,4). MEN 2B accounts for roughly 5% of MEN2 cases, and is characterized by MTC, pheochromocytoma (45%), ganglioneuromatosis (100%), and marfanoid habitus (65%) (4).

Since the introduction of *RET* proto-oncogene testing in 1995, the use of molecular techniques has allowed the members of affected families to receive a preclinical diagnosis of MTC, enabling disease prevention and early treatment (3). A wide range of *RET* mutations is classified according to the aggressiveness of MTC into three risk categories: highest-, high-, and moderate-risk mutations (5). Based on specific risk categories, the American Thyroid Association (ATA) developed follow-up and treatment recommendations, with an emphasis on prophylactic surgery in asymptomatic *RET* mutation carriers (4).

Despite the established mutation screening and counseling program, it is difficult to determine the exact incidence and prevalence of germline mutations within a population if molecular genetic information is not systematically collected and stored in a population-based nationwide registry (6). Even though some countries, such as Ireland, France, Norway, and Denmark, have comprehensive population-based databases, this is not a common practice in all European populations (7-10).

In the Slovenian population, numbering 2 063 077, the crude annual incidence rate of thyroid carcinoma reported by Cancer Registry of Republic of Slovenia (CRRS) is 9.9/100,000, with approximately seven new cases of MTC each year. The registry has been gathering data on all cancer types and issuing annual reports since 1950 (11). Genetic counseling and testing for potential *RET* mutation carriers has been offered since 1995 at the Institute of Oncology Ljubljana (OI), the national comprehensive center. In this article, we assessed the frequency and type of *RET* mutation in Slovenian MTC patient population diagnosed between 1995 and 2015 and estimated the crude annual incidence of MTC.

## Methods

The study involved Slovenian patients treated for MTC at the OI from 1995 to 2015 and their family members who took part in genetic counseling and testing at the same institution. Data were collected prospectively, and 192 cases were retrospectively reviewed. The time-period was defined based on the year of introduction of routine *RET* genetic testing in Slovenia and the availability of data in the CRRS. In Slovenia, genetic counseling and testing are offered to all patients diagnosed with MTC, and, if *RET* mutation is confirmed, to patients' first-degree family members.

**Figure 1:**
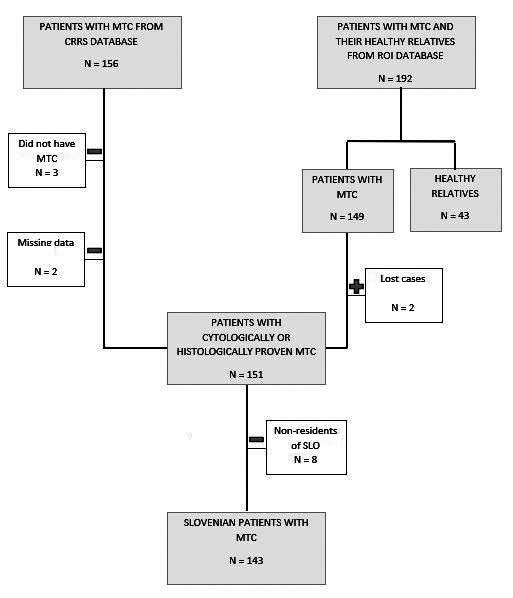
The total number of patients included in the analysis. Abbreviations: MTC – medullary thyroid carcinoma; CRRS – Cancer Registry of Republic of Slovenia; ROI – hospital-based Registry of Patients with MTC; SLO – Slovenia.

Two data sources were used: the hospital-based Registry of Patients with MTC (ROI) and the CRRS. The ROI included the epidemiologic and molecular genetic data from 149 patients with MTC and 43 relatives without proven MTC. CRRS included data from 156 patients diagnosed with MTC between 1995 and 2015. The study involved only the patients with MTC confirmed by histology or fine needle aspiration biopsy (FNAB). The patients with missing data and non-residents of Slovenia at diagnosis were excluded. The total number of patients with MTC and their relatives obtained after comparing the two sources was 186 (Figure 1).

The crude incidence rate was calculated as the number of new cases in one calendar year divided by the number of persons at risk of disease at the midpoint of the investigated period; it was expressed per 100 000 person-years (11). The data were obtained from the SI-STAT database, Statistical Office RS (12). The time trends, alongside the increase in the estimated crude incidence rate with annual percentage change, were estimated by joinpoint regression analysis using the Joinpoint regression program, version 4.6.0.0. (Statistical Methodology and Applications Branch, Surveillance Research Program, National Cancer Institute), which follows the models developed by Kim et al (13,14).

There were 51.6% of male patients. A total of 113 participants had MTC confirmed by FNAB before surgery. The majority of patients had a negative family history (113/186, 79.0%).

Among healthy relatives, 12 MTC (12/55) were discovered after prophylactic total thyroidectomy (TT). The number of relatives undergoing genetic testing was 2.8/proband.

There were 143 patients with MTC, either confirmed before genetic testing or discovered due to prophylactic TT, and 43 healthy relatives. MTC patients’ age at diagnosis and the mutation carriers’ age at the time of surgery was recorded, as well as treatment history of patients with MEN 2-associated diseases (Table 1).

**Table 1:** Characteristics of the study population

	All patients and their relatives included in the analysis, n (%)	Patients with MTC, n (%)	Healthy relatives without proven MTC, n (%)
**Total**	186	143	43
**Sex**			
female	90 (48.4)	72 (50.3)	18 (41.9)
male	96 (51.6)	71 (49.7)	25 (58.1)
**Family history**			
positive	64 (34.4)	21 (14.7)	43 (100.0)
negative	113 (60.8)	113 (79.0)	/
unknown	9 (4.8)	9 (6.3)	/
**MTC FNAB verified**			
yes	/	113 (79.0)	/
no	/	13 (9.1)	/
unknown	/	17 (11.9)	/
**Prophylactic TT**			
number	26 (13.9)	12 (8.4)	14 (32.6)
**MTC after surgery**			
yes	143 (91.1)	143 (100.0)	/
no	14 (8.9)	/	14 (100.0)
C-cell hyperplasia	52 (33.1)	40 (28.0)	12 (85.7)
**MEN 2 associated endocrinopathies**			
pHPTH	5 (9.8)	4 (10.8)	1 (7.1)
PHEO	9 (17.6)	9 (24.3)	0 (0.0)
HSCR	1 (2.0)	1 (2.7)	0 (0.0)

*Abbreviations: MTC – medullary thyroid carcinoma ; FNAB – fine needle aspiration biopsy; TT – total thyroidectomy; MEN – multiple endocrine neoplasia; pHPTH – primary hyperparathyroidism; PHEO – pheochromocytoma, HSCR – Hirschsprung’s disease.

After obtaining individuals' consent, a peripheral blood sample was taken. Genomic DNA was isolated from the peripheral blood leukocytes. Exons 10, 11, 13, 14, 15, and 16 of the *RET* proto-oncogene were amplified with polymerase chain reaction. Point mutations of the *RET* gene were detected by single-strand conformation analysis and DNA sequencing. The detected mutations were confirmed by restriction enzymes analysis. If the result was positive, conformation testing was performed using a new blood sample, as previously described (15). The mutation frequency was expressed as the number of families with a certain mutation. The number of tested individuals was expressed as a ratio of tested relatives/probands.

### Statistical analysis

The normality of distribution was assessed with the Shapiro-Wilk test. The data are expressed as frequencies and percentages for categorical variables and median with ranges for continuous variables, unless otherwise specified. Nonparametric data were compared with the Mann-Whitney U test. A *P* value of <0.05 was considered significant. Statistical analysis was performed with SPSS for Windows, version 19.0 (IBM Corp., Armonk, NY, USA).

## Results

**Figure 2:**
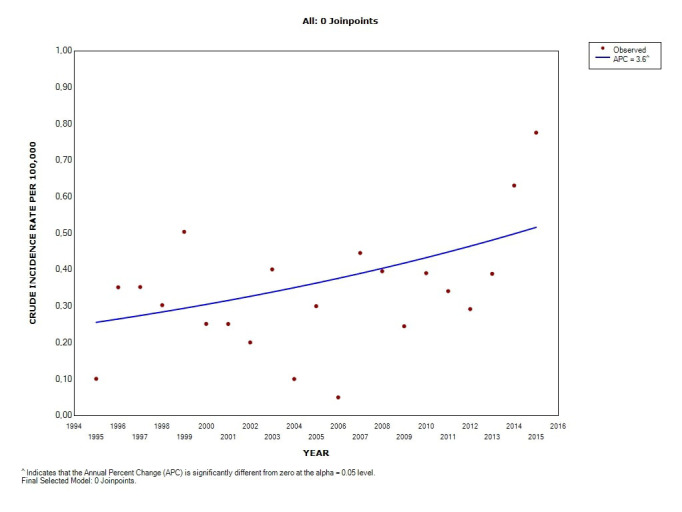
The crude annual incidence rates and long-term trends of incidence rates.

The crude annual incidence rate of MTC in the Slovenian population was 0.34/100.000, as 143 patients were diagnosed with MTC between 1995 and 2015. The estimated crude incidence rate increased significantly (*P* < 0.05), with annual percentage change (APC) of 3.6% (Figure 2).

Among 143 patients with MTC, 25.9% were mutation carriers (37/143). The patients belonged to 20 different families. *RET* mutation was discovered in all 21 patients with a positive family history (21/21) and in only 14.2% of patients with a negative family history (16/113). Among 9 patients with unknown family history, no mutation carriers were found. The remaining 85 (85/113; 75.2%) patients without a family history were *RET*-mutation negative, with the sporadic form of MTC. Two patients refused genetic testing (2/113; 1.8%). Benign variants were found in 10 (10/113; 8.8%) patients with MTC, who presented as sporadic cases (Table 2). The median age at MTC diagnosis was lower among *RET*-mutation positive patients than among sporadic cases (41.0 years vs 58.0 years, *P* < 0.05).

**Table 2:** Results of medullary thyroid cancer (MTC) patients and healthy relatives´ gene testing

	All patients and their relatives included in the analysis, n (%)	Patients with MTC, n (%)	Healthy relatives without proven MTC, n (%)
**Median age**			
*RET* positive	/	41.0	9.5*
*RET* negative	/	58.0^†^	/
**Gene test results**			
*RET* mutation positive	51 (27.4)	37 (25.9)	14 (32.6)
*RET* mutation negative	118 (63.4)	94 (65.7)	24 (55.8)
benign variants	15 (8.1)	10 (7.0)	5 (11.6)
testing refused	2 (1.1)	2 (1.4)	/
***RET* mutations per patient**			
C618F	8 (15.7)	7 (18.9)	1 (7.1)
C618S	7 (13.7)	4 (10.8)	3 (21.4)
C634R	4 (7.8)	3 (8.1)	1 (7.1)
C634G	2 (3.9)	2 (5.4)	0 (0.0)
C634Y	7 (13.7)	7 (18.9)	0 (0.0)
L790F	19 (37.3)	11 (29.7)	8 (57.1)
V804M	3 (5.9)	2 (5.4)	1 (7.1)
M918T	1 (2.0)	1 (2.7)	0 (0.0)

*Median age at the time of prophylactic total thyroidectomy (TT).

†*P* < 0.05 *RET* negative vs *RET* positive.

The mutation was most frequently found at codon 634 (6/20; 30.0%) and 618 (6/20; 30.0%), followed by mutations at codons 790 (5/20; 25.0%), 804 (2/20; 10.0%), and 918 (1/20; 5.0%). Two different intronic mutations and 4 different *RET* polymorphisms were detected (Table 3).

**Table 3:** Frequency of mutation and benign variants of *RET* proto-oncogene according to the number of families

Mutation	Exon	MTC risk level^†^	Total number of patients with MTC^‡^	No. of families	Positive family history	Negative family history	No. of healthy relatives, who are *RET* mutation or BV carriers	MTC /No. of prophylactic TT
**C618F**	10	MOD	7	4	3	4	2	1/2
**C618S**	10	MOD	4	2	4	0	5 + 1 BV	2/5
**C634R**	11	H	3	3	1	2	1	0/1
**C634G**	11	H	2	1	1	1	0	0/0
**C634Y**	11	H	7	2	6	1	4	4/4
**L790F**	13	MOD	11	5	6	5	13	5/13
**V804M**	14	MOD	2	2	0	2	1	0/1
**M918T**	16	HST	1	1	0	1	0	0/0
**S649L**	11	BV	1	1	0	1	0	0/0
**G691S**	11	BV	2	2	0	2	0	0/0
**S836S**	14	BV	2	2	0	2	0	0/0
**S904S**	15	BV	2	2	0	2	0	0/0
**c.1648 + 84G>A, c.1648 + 88delC**	int.8	BV	2	2	0	2	3	0/0
**c.1648 + 84G>A, c.1648 + 88delC, S836S**	int.8 14	BV	1	1	0	1	1 1	0/0

*Abbreviations: MTC – medullary thyroid carcinoma; BV – benign variants; TT – total thyroidectomy; HST – highest risk; H – high risk; MOD – moderate risk; GC – gene carriers.

†MTC risk levels (5).

‡35 patients with histologically proven MTC after therapeutic TT and 12 patients with histologically proven MTC after prophylactic TT.

## Discussion

The crude annual incidence rate of MTC in Slovenian population was 0.34/100,000. We identified significant annual incidence changes and quantified the incidence trends (16). Over the 21-year period, the MTC incidence in the Slovenian population increased by approximately 3.6% annually, which represents a significant increase. The crude annual incidence rate in the Slovenian population is approximately three times higher than that reported in Ireland (0.11/100,000) and higher than that reported in Denmark (0.28/100,000) (8,17). The difference could be attributed to a different incidence of thyroid malignancies in general and different age distribution in the population. For example, the Irish population is on average younger than the Slovenian population (median age in 2016: 37.4 vs 42.9 years) (12,18).

To estimate the disease incidence and the prevalence of the germline mutations, it is necessary to systematically collect patients’ and molecular genetic information. The use of data obtained from the CRRS, one of the oldest population-based registries in Europe, enabled us to more precisely evaluate the cancer incidence in the population than we would be able using only hospital-based registries, which serve as a base for most other research reports (19). Many researchers depended on single-center or multi-center patient data, which do not always reliably represent the nationwide population (20,21).

To our knowledge, six population-based analyses have been published so far (7-10,17,22). A Norwegian study (10) described all known *RET*-positive MEN 2A patients with MTC from four centers. A French study (9) investigated the spectrum of *RET* benign variants based on data from all centers performing *RET* analysis. These two studies, although nationwide, did not involve all the patients with MTC diagnosed in the defined period (9,10). Conversely, researchers from Ireland retrospectively analyzed all newly diagnosed patients with MTC, using data from the Irish National Cancer Registry, while two studies from Denmark used data from three centers covering the whole Danish population (7,8,22). A recent study from Denmark used data from three nationwide registries (17). To the best of our knowledge, our study is the first to compare data from a hospital-based registry with those from a national cancer registry, an approach that also evaluates the reliability of the hospital-based registry.

In our study, 25.9% of patients with MTC harbored a germline *RET* mutation. The result is in accordance with other published reports, where the prevalence of inherited MTC was 20%-25% (2). The mutations were discovered in exons 10, 11, 13, 14, and 16, while exons 8 and 9 were not tested in the investigated period. The most frequent mutations were *RET* germline mutations of codons 634 and 618 (30.0%), and exon 11 was the most frequently altered exon. Codon 634 mutations have been reported as the predominant mutations in several European and non-European studies (4). This findings might be explained by an earlier disease onset and the presentation of a full-blown MEN 2 syndrome (MTC, pheochromocytoma, and primary hyperparathyroidism) (7). Mutations of codons 790, 804 and 918 were observed in a smaller percentage of patients (25.0%, 10.0% and 5.0%).

In our population, the frequency of L790F mutations was 25% (4). A large German study reported L790F mutations in only 13% of cases, while numerous other studies did not detect any codon 790 mutations (6). On the other hand, the V804M mutation in exon 14, the most frequent *RET* mutation in Italy, was found in just two of our patients (21). The population-specific variety of *RET* mutations has already been reported by some authors (7,20,23,24).

In our study, an M918T mutation, associated with MEN 2B, was discovered in exon 16 in an eight-year-old patient. This mutation occurs in more than 95% of patients with MEN 2B syndrome and is, due to the early onset of MTC and an aggressive form of the disease, classified as a highest-risk mutation. In 95% of patients with MEN 2B, the germline *RET* mutation occurs *de novo*. Although children with *de novo RET* mutations are usually recognized because of symptomatic MTC or pheochromocytoma, our patient was discovered as a *de novo* M918T mutation carrier due to nonendocrine disease manifestations (intestinal neuronal dysplasia, tetralogy of Fallot, hypothyroidism, bilateral pyelectasis, short stature, and hypermobility syndrome) (5). MTC was confirmed later during the course of diagnostics, and TT was performed.

Specific *RET* mutations create a particular phenotype and affect the clinical course of MTC, with a strong genotype-phenotype correlation (25). In our patients with MTC, the mutations on the cysteine-rich extracellular domains of the tyrosine kinase receptor were associated with unilateral or bilateral pheochromocytoma in 39.1% and with primary hyperparathyroidism in 17.4% of cases. The prevalence of MTC-accompanying endocrinopathies observed in our study is in accordance with that reported in other studies and ATA guidelines (5).

Pheochromocytoma is rarely the first tumor to be diagnosed in *RET* carriers, as it usually presents in the third or fourth decade of life (25). In six of our patients, pheochromocytoma was recognized before the diagnosis of MTC, with the median age at diagnosis of 37 years. Genetic screening revealed a codon 634 mutation in all patients. All patients diagnosed with pheochromocytoma are recommended to undergo genetic counseling and screening (26,27), which enable the identification of other carriers in the same family and allow increased perioperative surveillance of the patient (27,28).

Among the families investigated in our study, one patient with a C618S mutation in exon 10 had MTC and Hirschsprung’s disease. The coexistence of MTC with Hirschsprung’s disease is rare and is usually associated with mutations of codon 620 and, to a lesser extent, of codons 609, 611, and 618 (15,21). Patients with these double-functioning mutations, due to their ability to be simultaneously activating and inactivating, should be monitored for Hirschsprung’s disease development. Conversely, screening of exon 10 is recommended in all individuals with Hirschsprung’s disease (20).

Some authors proposed that the clinical characteristics of the MEN 2A syndrome are modified by specific polymorphic variants or haplotypes of the *RET* gene (29). We detected benign variants in 10 MTC patients who had no pathogenic *RET* mutation, while no benign variant was confirmed in the *RET* mutation carriers. Although the result is intriguing, the importance of benign variants discovered in our study has yet to be confirmed.

Since exons 8 and 9 were not tested in our population, it is interesting to note that researchers from Ireland in 2013 reported on a Slovenian patient with the G533C mutation as the first case of a *RET* mutation in exon 8 (20,30). The described mutation is specific for the Mediterranean region and has rarely been found in other populations. The rarity of reports on G533C-positive families outside of Western Europe could be explained by seldom inclusion of exon 8 in routine screening (20). The recently implemented next-generation sequencing is expected to provide information about exons 8 and 9 in future MTC patients.

Despite the small population of patients with MTC in our study, the high frequency of mutations in the moderate risk category, and prophylactic TT performed relatively late in life, MTC patients with sporadic and hereditary form of the disease significantly differed in age. A similar observation has been previously reported. Sporadic MTC patients are usually diagnosed at significantly older age as the tumor is not noticed until the palpable thyroid enlargement and/or cervical lymphadenopathy is evident (31,32). At the other end of the spectrum are individuals with hereditary MTC discovered almost exclusively during family screening, before the clinical evidence of the disease appeared (33).

*RET* germline mutations are quite commonly discovered even in the apparently sporadic form of the disease. In our population, the mutation was discovered in 14.2% of patients with a negative family history. One would assume that the majority of seemingly sporadic patients carry low-risk mutations associated with a later age of onset of MTC and a less aggressive clinical course (8,21,34). Surprisingly, our genetic testing identified 4 patients with a high-risk mutation at codon 634 and 11 patients with a moderate-risk mutation. Apparently, according to some researchers, sporadic MTC leads to the identification of the familial disease in 3-10% of cases (8,21,34,35). An even greater prevalence has been reported in Turkey and Iran, 10.7% and 17.6% respectively (36,37).

The reasons behind the unrecognized familial diseases may be unknown family history or variable disease penetrance. Another possible reason could be limited disclosure of information about the hereditary nature of the disease to other family members due to cultural reasons, guilt or fear, and lack of education about the importance of family genetic counseling and testing (3). The presence of germline *RET* mutation in 14.2% of apparently sporadic MTC cases reinforces the need for *RET* genetic screening in all patients with MTC (21).

The communication about the hereditary form of the disease among family members is at least partially reflected in the number of tested individuals from a certain family. In our study, 2.8 relatives/proband were tested. The number is somewhat lower than expected (20,21). A better compliance was observed in an Italian study, with 3.4 relatives/proband, while a slightly lower compliance was observed in a Greek study (2.2 relatives/proband) (21). The results do not considerably differ from those obtained in publicly more recognizable *BRCA* mutation testing. A recent Spanish study on *BRCA* mutation testing reported 3.6 tested relatives/proband, similarly to the Italian report on *RET* families (38). However, a much higher compliance was observed in Denmark, with 8.9 relatives/proband (7). Interestingly, Turkish researchers reported that the frequency of mutation carriers among apparently sporadic MTC decreased from 10.7% to 5.2% due to increased awareness and genetic testing among physicians. Unfortunately, their limited efficiency could be observed in the number of performed prophylactic procedures (10/24; 41.7%) (39). The observed differences in the compliance of healthy relatives may indicate social and ethical differences in the attitude toward genetic counseling and screening (40).

The main limitations of our report are the low compliance of family members, resulting in a small number of tested individuals, and a lack of information about exons 8 and 9, which may have underestimated the frequency of *RET* mutation in the Slovenian population.

In conclusion, in a 21-year period the crude annual incidence of MTC in Slovenia was 0.34/100,000, with 3.6% annual increase. The analysis of national data revealed that 25.9% of Slovenian patients with MTC were RET mutation carriers. The most frequently altered codons were 634 and 618, but 25% of codon 790 mutations were also discovered. Annual incidence increase and nation-specific frequency of discovered RET mutations justify the continuation of gene counseling and testing of MTC patients in Slovenia.
